# Formation of metal/semiconductor Cu–Si composite nanostructures

**DOI:** 10.3762/bjnano.10.240

**Published:** 2019-12-13

**Authors:** Natalya V Yumozhapova, Andrey V Nomoev, Vyacheslav V Syzrantsev, Erzhena Ch Khartaeva

**Affiliations:** 1 Buryat State University, Smolina str., 24a, Ulan-Ude 670000, Russia; 2 Institute of Physical Materials Science, Siberian Branch of the Russian Academy of Sciences, Sakhyanovoy str., 6, Ulan-Ude 670047, Russia

**Keywords:** composite nanoparticle, gas-phase synthesis, molecular dynamics modelling

## Abstract

Molecular dynamics modelling of the formation of copper and silicon composite nanostructures was carried out by using the many-particle potential method. The dependences of the internal structure on the cooling rate and the ratio of elements were investigated. The possibility of the formation of the Cu–Si nanoparticles from both a homogeneous alloy and two initial drops at short distance were shown. A comparative analysis showed that the diameter distribution of copper and silicon atoms in experimental particles coincides with the simulation results with silicon content of 50 atom %. Additionally, an estimation of the effective experimental cooling rate was made.

## Introduction

In recent years, due to the development of various methods of synthesis, it has become possible to create composite Janus-like or core–shell nanoparticles. The size and the unique morphology at the nanoscale provide new optical, electronic, magnetic and surface properties to these nanoparticles [1–2]. In addition, it is possible to combine materials of which alloys do not exist in nature. The shell can be oxides, noble metals, phosphates or polymers [3–4]. Compositions of materials that are immiscible in the bulk state, such as Mo–Cu [5], Ag–Si [6], Ag–Cu [7], Au–Ni [8], are of special interest. Shells can have both a smooth spherical or a polyhedral shape, can have mesopores [9] or dendrites [10], or can even consist of three layers [11–12]. These variations can be useful for practical applications, e.g., for enhancing the absorption capacity of particles or for plasmon–exciton interaction [13].

The presence of an inorganic shell on a metal particle often leads to a significant improvement of the thermal stability of the core and, under the condition of a hermetic coating, reliably protects its surface from redox reactions [3]. Janus-like metal/semiconductor nanoparticles are promising as effective radio-absorbing media and are the basis for creating the elemental basis for microwave electronics and radiophotonics due to their large dipole moment and the appearance of a Schottky barrier in the contact region [14].

The structure of these nanoparticles is primarily determined by the methods and conditions of synthesis, which should allow us to combine the two materials even if they are immiscible in the bulk state. In addition to chemical techniques [9–12], physical methods such as gas-phase methods [5–6,15], laser ablation [7–8,16], and magnetron-sputter gas-phase condensation [17] have been developed. When these methods are combined with the possibility of rapid heating and evaporation of both materials and the possibility of rapid controlled cooling of the resulting vapour mixture, the formation of nanoparticles with a complex structure is possible.

In [18–21], the mechanism of formation of core–shell and Janus-like nanoparticles based on the difference in surface energies of the components and the thermodynamic analysis of two-component phase diagrams was investigated. However, this mechanism does not take into account the nonequilibrium of the formation process (metastable states) that may occur under different synthesis conditions. The extremely fast heating leads to a high kinetic energy of atoms, overcoming the repulsion force between immiscible atoms. As a result of their mutual diffusion and fast cooling/solidification, the atoms may remain in an intermixed state [8]. Therefore, the role of the kinetic characteristics (cooling rate and residence time in the liquid phase) in the formation, structure, and morphology of nanoparticles remains unclear.

In [17], the simulation of the formation of the Si/Au core–shell nanoparticles obtained from two liquid drops of gold and silicon was presented. Additionally, the authors, using the Mie scattering theory, investigated the optical response of the obtained Si/Au nanoparticles. The results of the study showed an increase of the local electric field and unidirectional light scattering with a high Purcell coefficient compared with a nanoparticle consisting only of gold.

Another approach to modelling the formation of metal/semiconductor core–shell nanoparticles was described in [18]. There, a core–shell particle was obtained by spraying the outer shell on an already formed core. The molecular dynamics calculation of such a procedure showed the possibility of the formation of Cu–Si core–shell nanoparticles upon the condensation of silicon atoms onto the core when a copper nanocluster is introduced into a gaseous medium consisting of silicon atoms. In [22], similar particles were obtained by laser ablation of Au nanoparticles onto larger Co-oxide particles and agglomeration with a quasi-core–shell structure. In [23], the first results on modelling the formation of core–shell nanoclusters consisting of copper and silicon atoms in a melted state have been presented.

In this paper, we have proposed novel principles of formation: 1) a core–shell structure made of a silicon/copper liquid alloy and 2) core–shell and Janus-like nanoparticles made of liquid silicon and copper droplets.

We have presented new data on the formation of metal/semiconductor nanoclusters, such as the transition of particles from a core–shell structure to a Janus-like structure starting from the liquid state, depending on the cooling rate, silicon content, and the distance between the components.

In order to identify and refine the ways of formation of two-component nanoparticles at the atomic level, we experimentally obtained the core–shell nanoparticles, Janus-like nanoparticles, and modelled the formation of nanoclusters of various morphologies.

## Experimental Technique

**Figure 1:**
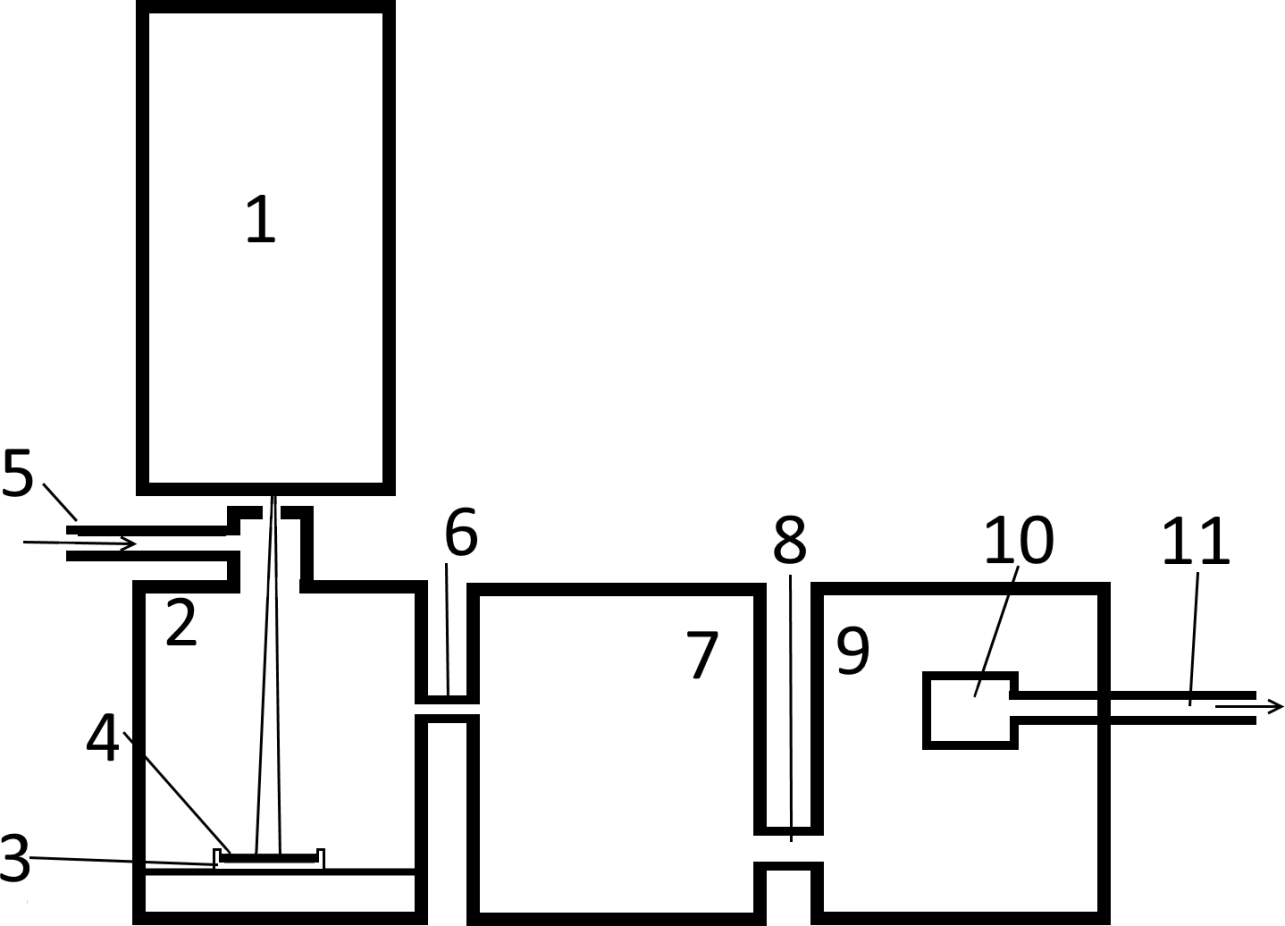
Diagram of the installation for the production of composite nanoparticles. Elements of the installation: 1 – linear electron accelerator ELV-6; 2 – evaporation chamber; 3 – graphite crucible; 4 – evaporated materials; 5 – input pipe for the transport gas; 6 – connecting pipe; 7 – intermediate chamber for cooling the aerosol and partial deposition of the powder; 8 – connecting pipe; 9 – filter chamber; 10 – filter; 11 – pumping nozzle.

We used an installation based on a relativistic electron accelerator with an energy of 1.4 MeV and a current in the range of 5–25 mA as the heating source (Figure 1) to obtain composite nanoparticles. At the first step, the electron beam from the accelerator (1) irradiates the surface of the source materials (4), which are located in a graphite crucible and consist of two mixed solids. The current has a small value of 5–10 mA. As a result, the source materials are transformed into a liquid state. Then, in the second stage, the beam current rises up to 20 mA, and the mix begins to evaporate from the surface. The transport gas comes through the nozzle (5), enters into the evaporation chamber (2), and cools the vapours of two substances and transfers them into the intermediate chamber (7) through a nozzle (6) for cooling the vapours and partial precipitation of the powder. The composite nanoparticle vapour, passing into the chamber (9) through the pipe (8), is deposited on the filter (10).

## Simulation Technique

For the simulations, we used the molecular dynamics method, in which the matter is considered at the atomic level without explicitly taking into account the electronic subsystem, and the interaction between atoms was determined by semi-empirical potentials based on the immersed atom method (EAM potentials) [23]. It was successfully used for modelling the processes of condensation of metal nanoparticles and nanoalloys based on them. In this study, the simulation of thermal effects on CuSi nanoclusters was carried out using the modified immersion atom potentials (MEAM) [24]. For the numerical integration of the motion equations, the Verlet algorithm was used, and the time step was τ = 1 fs. The simulation was carried out in the LAMMPS molecular dynamics research package [25]. A part of the calculations was performed using the resources of the Information and Computing Center of the Novosibirsk State University.

## Results and Discussion

### Modelling a free spherical cluster of a copper/silicon alloy

We considered a free spherical cluster of a copper/silicon alloy with a random distribution of atoms of 4.6 nm in size, with different silicon concentrations, obtained by cutting a sphere from an ideal fcc lattice as the initial object. Initially, the cluster was in the liquid state.

The beginning of the cooling process was accompanied by the relaxation of the initial state at a temperature *T* = 1800 K. The cluster was cooled to a temperature of 300 K within the framework of the canonical ensemble using the Nose thermostat. The time step was τ = 1 fs. The cooling rate is 1 K/ps (10^12^ K/s). For the calculation, we used the speed variant of the Verlet algorithm. The visualisation and analysis of the simulation results was carried out in the OVITO program [26–27].

**Figure 2:**
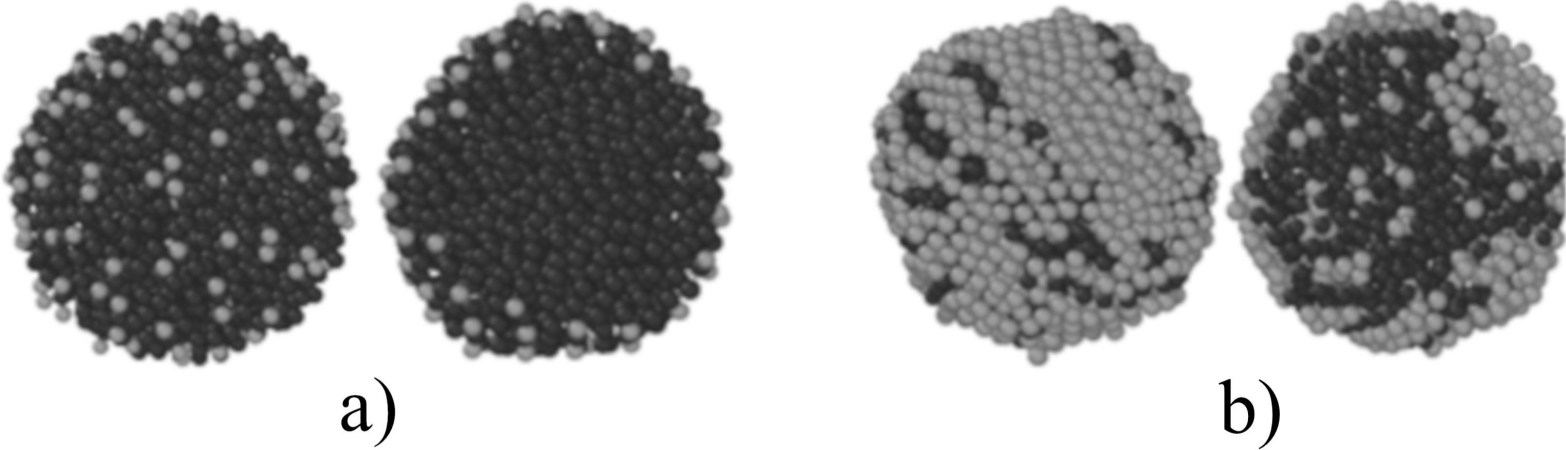
Clusters with different silicon contents after 1.5 ns (cooling rate 1 K/ps); dark atoms are copper, light atoms are silicon (on the left is the appearance, on the right is in the incision): a) 10 atom %; b) 50 atom %.

Figure 2 shows clusters with different silicon content at 1.5 ns after the start of simulation. As revealed in [23], with a low silicon content of 10 atom %, a core–shell structure is formed (Figure 2a). The simulated system seeks to minimise the surface free energy. Therefore, because the surface tension of copper is greater than that of silicon, the silicon atoms are segregated to the surface of the cluster, and copper atoms are segregated to the cluster core. The nucleus formed consists mainly of copper atoms and a small number of silicon atoms. However, after increasing the silicon concentration up to 50 atom %, silicon atoms are observed in large quantities in the centre of the core–shell particle (Figure 2b). A similar interconnection of the composition of the alloy and its structure, in particular, the size and phase composition of the nanoparticles has been presented in [5].

**Figure 3:**
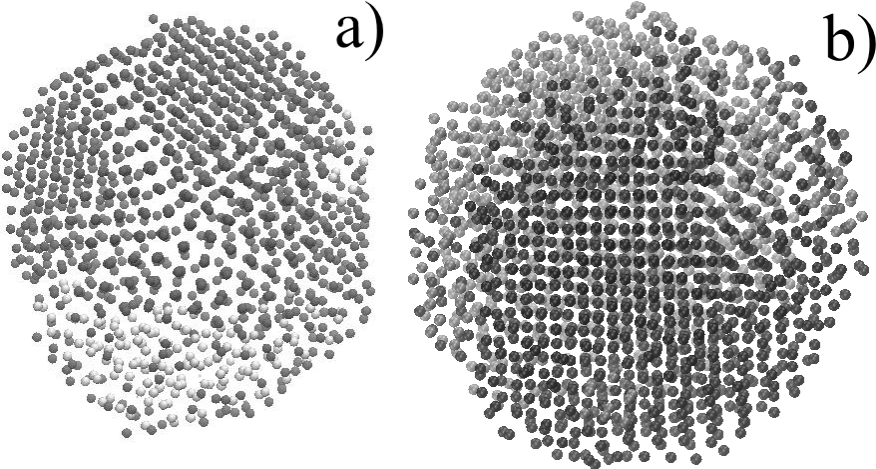
The structure of Cu–Si clusters: a) cooling rate of 1.5 K/ps (IR and DK phases); b) the cooling rate is 1 K/ps (fcc and hcp phases). Light grey: silicon, dark grey: copper.

The analysis of the obtained data on atomic dynamics in nanoscale clusters showed that at a cooling rate of 1.5 K/ps, a core–shell structure is formed in 70% of the clusters and the Janus-like structure is formed in 30% of the clusters. In the obtained cluster structures, twinning prevails as a mixture of the icosahedral (IR) and dodecahedral (DK) phase (Figure 3a). With a decrease in the cooling rate to 1 K/ps, the probability of the formation of the Janus-like structure increases to 50%. In addition to a change in morphology, a change in phase is also observed. In this case, the fcc phase is adjacent to the hcp phase (Figure 3b).

These results are consistent with the simulation of the formation of copper nanoclusters [14] as well as the synthesis of composite AuNi nanoparticles in [8]. This is explained by the fact that with a decrease in the cooling rate, the atoms retain a high mobility. This results in both the silicon atoms replacing the copper atoms in the crystal lattice of the cluster and a sufficient time for the separation (mobility) of the atoms during the cooling process. A variation of the cooling rate results in large variations of the structure of the final nanoparticles [5], in particular of their phase.

### Modelling of silicon and copper droplets

**Figure 4:**
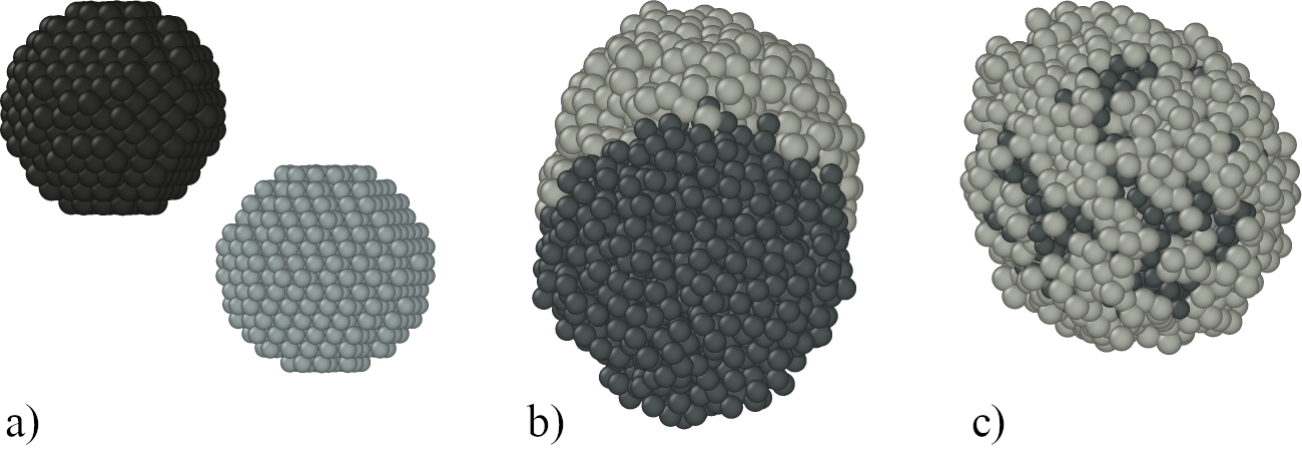
Changes in the structure of the nanocluster in time: a) initial state; b) intermediate Janus-like structure; c) core–shell structure.

Here, we have considered the case that the initial objects were liquid droplets of silicon and copper, 1.2 nm in size, located at a short distance from each other (Figure 4a). The initial temperature of the droplets is *T* = 1800 K. The value of the initial temperature was chosen above the melting points (copper melting point *T*_Cu_ = 1356.55 K, silicon melting point *T*_Si_ = 1688 K) in order to destroy the long-range bonds in the materials.

Cooling to a temperature of 300 K was simulated with a cooling rate 1.5 K/ps. A slight diffusion of liquid silicon into liquid copper began immediately after the start of the simulation. Then, over a period of a few nanoseconds, the drops of copper and silicon approached each other and a Janus-like structure was formed (Figure 4b). Furthermore, after the next few nanoseconds, a core–shell nanocluster was formed.

It was noted that when the silicon content was low, the segregation of silicon atoms to the cluster surface occurred almost immediately after the cooling started, and, in most cases, the cluster did not change the core–shell structure. At the same time, for a silicon concentration of equal or more than 50 atom %, the segregation process largely depended on the cooling rate.

**Figure 5:**
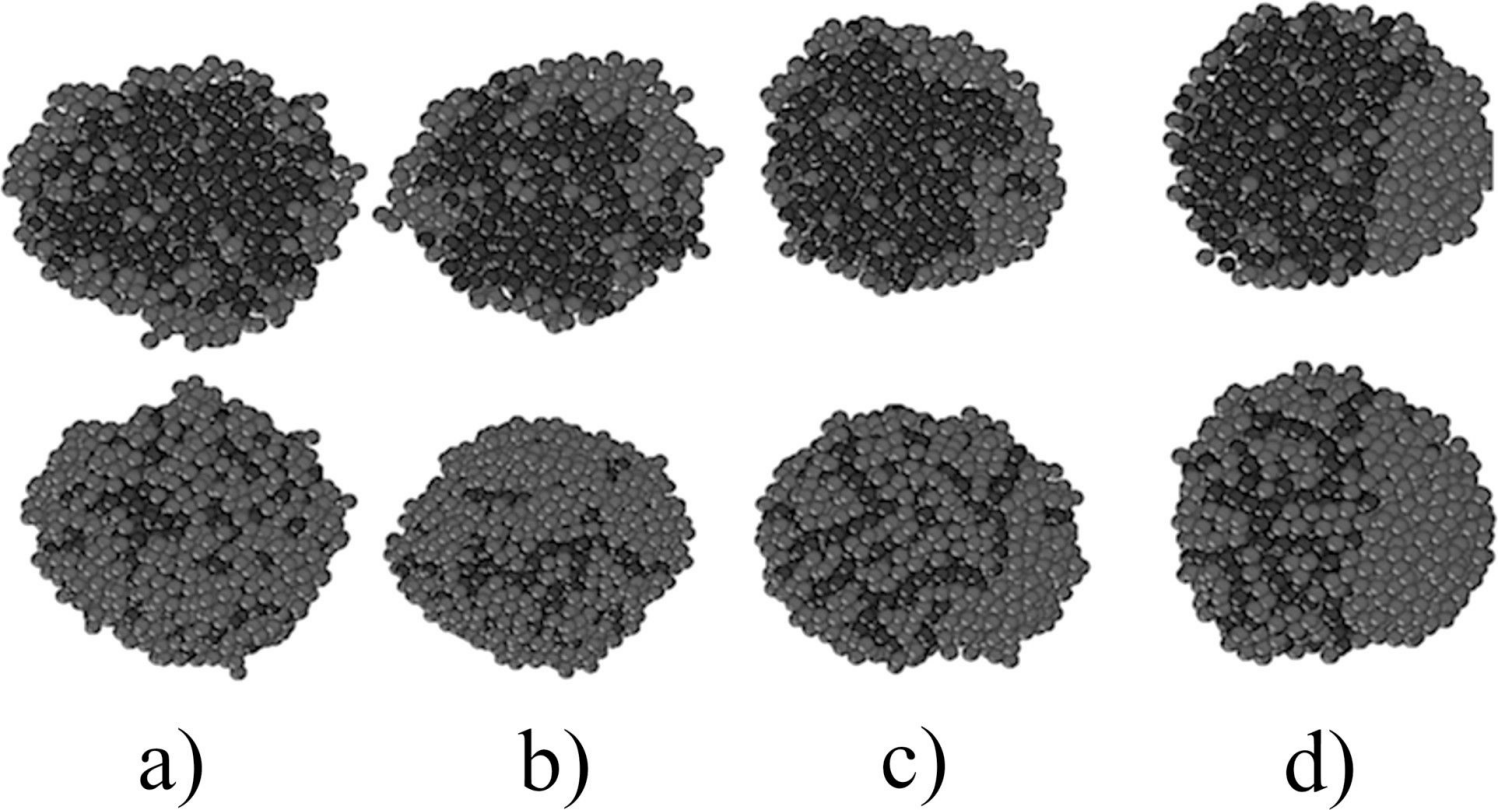
The structure of a Cu–Si nanocluster with a silicon content of 50 atom % at different times at a cooling rate of 0.002 K/ps after a) 0.75 ns, b) 7.5 ns, c) 75 ns, and d) 750 ns; top row: cross section, bottom row: outside view.

The simulation of a nanocluster with a size of 4.6 nm containing 50 atom % silicon and copper atoms, with the initial state of two drops, showed the following: A significant decrease in the cooling rate to a value of 0.002 K/ps leads to the fact that copper and silicon from the core–shell structure begin to segregate, thereby striving to form a Janus-like nanocluster. Figure 5 shows a consistent change of the nanocluster structure at different time points. Furthermore, it shows a gradual modification of the homogeneous distribution of atoms in the melt into a Janus-like structure.

The first reason for such a transformation is the large thickness of the silicon shell at 50 atom % content of both components. The reason of the formation of such a particle is the difference in the surface energy between the silicon and copper components. It becomes insignificant, especially for silicon particles located at large distance from the central copper part. The second reason is that silicon has a higher cohesive energy (4.63 eV) than copper (3.54 eV).

### Experimental results

**Figure 6:**
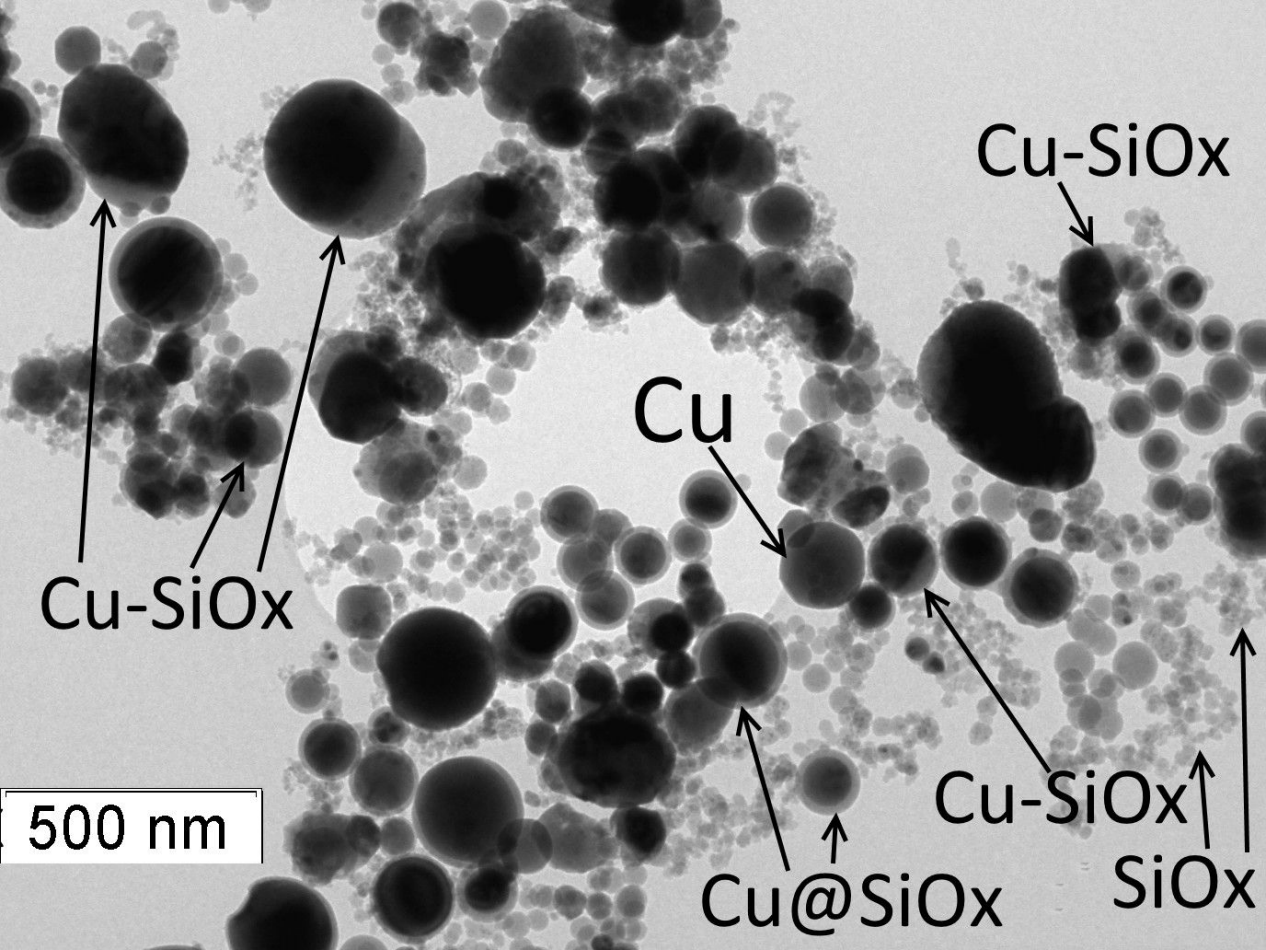
Transmission electron microscopy of the Cu–SiO*_x_* Janus-like, Cu@SiO*_x_* core–shell, pure Cu and SiO*_x_* nanoparticles (1 < *x* < 2).

**Figure 7:**
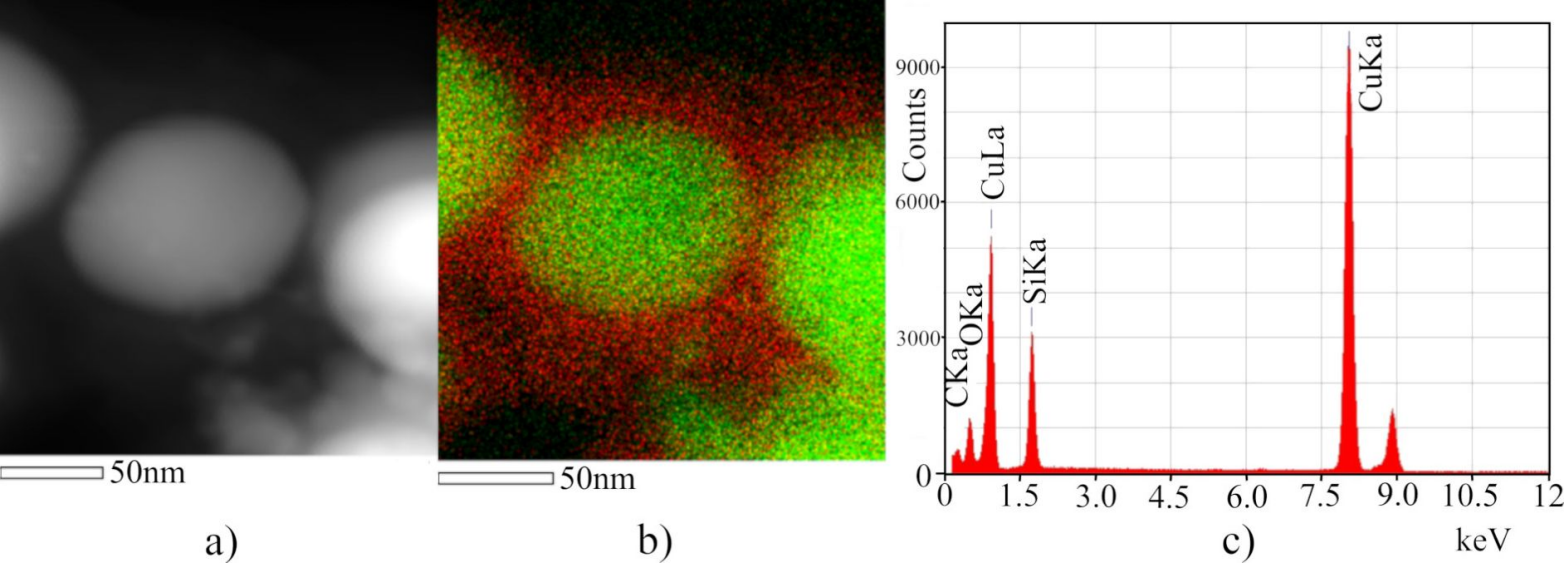
a) Scanning electron microscopy image of the obtained Cu@SiO*_x_* nanoparticles (1 < *x* < 2); b) elemental mapping, dark (red): silicon, light (green): copper; с) EDX-spectrum.

Experimentally obtained Cu–SiO*_x_* Janus-like and the Cu@SiO*_x_* core–shell composite nanoparticles from evaporating the raw materials with a relativistic electron beam are shown in Figure 6 and Figure 7. The average size was obtained from the particle size distribution of 500 nanoparticles of each certain type. The average size of the core–shell particle does not exceed 100 nm, as observed from transmission electron microscopy images (Figure 6, Figure 7a). The results of the elemental mapping of nanoparticles (Figure 7b) shows that copper and silicon are concentrated in the core and on the surface of the particle, respectively. The X-ray fluorescence analysis (EDX) of Cu–SiO*_x_* nanoparticles, taken on a section of size 500 × 500 nm (Figure 7c), confirms that the particles consist of copper, silicon, and oxygen. The nanoparticles are stable retaining their properties for several months.

The transmission electron microscopy image shows core–shell particles, Janus-like nanoparticles, and individual particles SiO*_x_* (Figure 6). The modelling of the nanoparticle formation from the original nanoscale droplets described above suggests that Cu–SiO*_x_* particles can be formed from copper and silicon droplets at a slow cooling rate. The oxidation is most likely to occur after the formation of a silicon cluster.

The EDX analysis along the middle line of the Cu–SiO*_x_* nanoparticle provides the distribution of elements in more detail (Figure 7b). The copper content is about 75 atom %, that of silicon and oxygen is about 25 atom % with approximately equal proportions in the centre of the nanoparticle. An increase in the copper content at a distance of more than 115 nm is obviously due to the presence of a neighbouring particle (Figure 7a,b).

**Figure 8:**
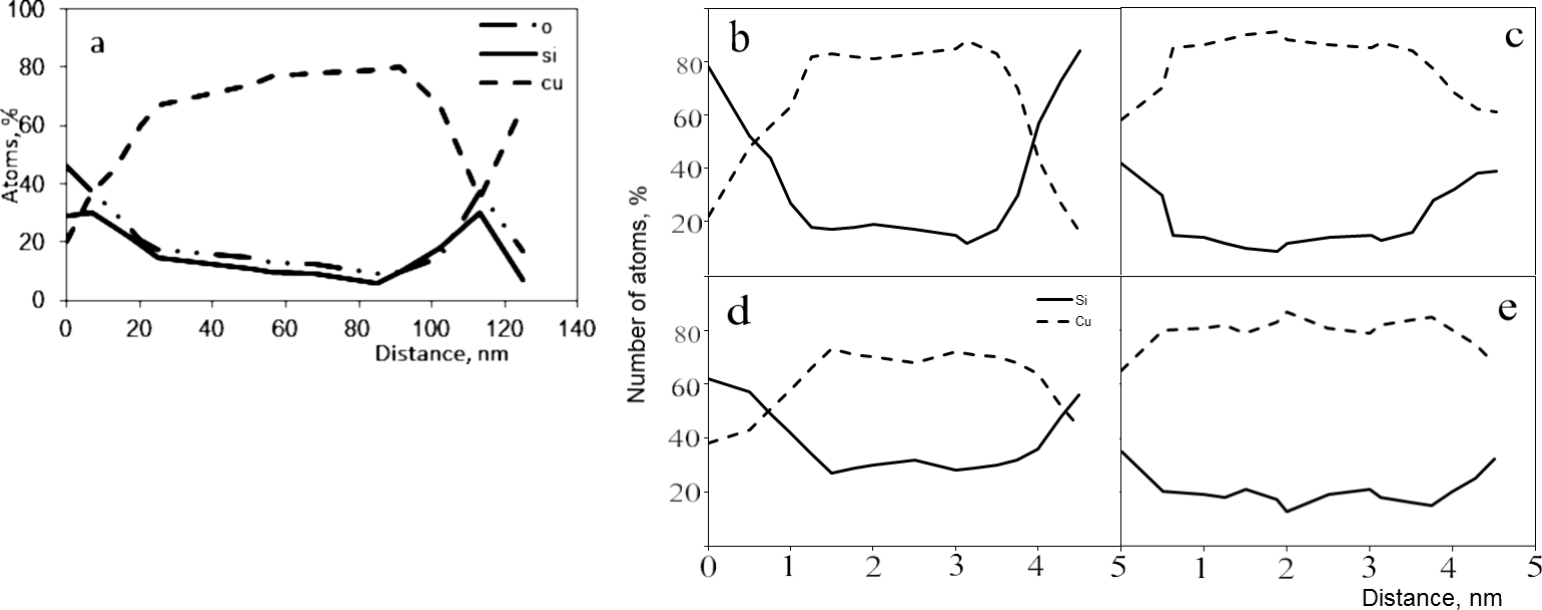
The distribution of the elements copper, silicon, and oxygen along a line passing through the centre of the nanoparticle; a) experimental result; b–e) modelling results with cooling rates of b) 1 K/ps, 50 atom % silicon, c) cooling rate 1 K/ps, 10 atom % silicon, d) cooling rate 1.5 K/ps, 50 atom % silicon), e) cooling rate 1.5 K/ps, 10 atom % silicon.

A clearer image of the result of the EDX analysis is shown in Figure 8a. It shows that in the central region of the particle, the copper content varies from 70 to 80 atom % and the silicon content from 10 to 20 atom %. At the periphery of the particle, the copper content is significantly reduced, while that of silicon and oxygen increases. It should be noted that the concentrations become equal at around 40 atom %. The width of the transition region is about 20 nm, which can be estimated as the thickness of the shell of the nanoparticle, where the concentration of the elements is aligned.

Figure 8 shows a comparison of EDX analysis of experimental and model nanoparticles obtained from the liquid phase for a cooling rate of 1 and 1.5 K/ps with a silicon concentration of 10 or 50 atom %. The values are obtained by summing the number of atoms in the cross section perpendicular to the axis of the nanoparticle and corresponds to the principle of the EDX analysis.

In Figure 8c, the copper content is about 85 atom % in the centre of the nanoparticle. As the cooling rate increases to 1.5 K/ps, the copper content in the centre of the nanoparticle decreases to 80 atom %, with an increase in the silicon content (Figure 8e). Note the more gradual decrease in concentration in the transition region. At a cooling rate of up to 1.5 K/ps, the shell thickness can be estimated at 10–12 nm, and at a cooling rate of 1 K/ps (Figure 8c), it can be estimated at 5–6 nm.

In the modelled nanoparticles with a silicon concentration of 50 atom % (Figure 8b,d), the distribution is more similar to that obtained in the experiment (Figure 8a). In this case, the effect of the cooling rate on the particle distribution is more pronounced. Additionally, at a low cooling rate, the copper content in the core is about 85 atom % (Figure 8b) with low silicon content (Figure 8c). However, at a high cooling rate, the copper content in the core decreases to 70 atom %. This indicates that the increase in the cooling rate “freezes” silicon in the core of the nanoparticle.

According to the initial data in the experiment, the content of silicon and copper was the same. Therefore, direct comparison of the data in Figure 8a,b and in Figure 8d is possible. Based on these data, it can be assumed that the cooling rate in the experiment can be estimated at 1.25 K/ps.

A direct measurement of the cooling rate in the experiment is impossible due to the high temperature of the sample in the evaporation chamber under irradiation by a relativistic electron beam. A rough estimate of the cooling rate based on the experimental parameters is 10^5^ K/s, which is significantly less than the modelled value of 10^12^ K/s. This is most likely due to the strong nonequilibrium of the evaporation–condensation processes. However, the presented comparative data on the cooling rates in the experiment and simulation allow us to estimate the ratio between them, which may be important regarding its use both for experiments concerning the synthesis of composite nanoparticles and for fundamental science.

The formation of core–shell particles, under the conditions of the experiment, when a liquid two-component melt is preliminarily created before evaporation, most likely occurs when condensed copper and silicon drops merge and segregate due to the difference of their surface energies. The appearance of oxygen in the compound with silicon is due to its presence in copper and the oxidation of silicon in air after production.

The calculations and the comparison with experiments demonstrate that it is possible to obtain composite nanoparticles with different content and distribution of components. This is indicative of the possibility of producing composite nanoparticles with various properties, which could be useful in various applications. The strong nonequilibrium processes make it possible to obtain composite nanoparticles from elements immiscible under standard conditions [5–8]. The basic parameters, as was shown here, are the ratio of the initial materials and the vapour cooling rate, which also determine the zone of particle formation and their size. Variation of these parameters under the conditions of a local synthesis setup does not require significant efforts, but it creates the possibility of obtaining composite nanoparticles with unique properties.

## Conclusion

The simulation results show that the creation of a Cu–Si core–shell nanoparticles from a liquid phase of randomly distributed silicon and copper atoms in a lattice with 10 atom % silicon content is possible at a cooling rate of 1 K/ps.

The formation of Janus-like nanoparticles of two liquid contiguous nanoclusters occurs at lower cooling rates compared to core–shell nanoparticles if the silicon content is 50 atom %.

A significant decrease in the cooling rate to a value of 0.002 K/ps results in the core–shell structure beginning to separate and forming a Janus-like nanoclusters. The results of the experiments and the computer simulations allow for the conclusion that the internal structure of the composite cluster depend on the type of initial objects, silicon concentration in the alloy and cooling rate. Furthermore, the effective cooling rate of the Cu–SiO*_x_* core–shell nanoparticles experimentally obtained by the electron beam evaporation method has been estimated.

## Funding

This work was supported by the Russian Scientific Foundation [project number 18-79-10143].
